# Introducing electronic monitoring of disease activity in patients with chronic inflammatory demyelinating polyneuropathy (EMDA CIDP): trial protocol of a proof of concept study

**DOI:** 10.1186/s42466-023-00267-3

**Published:** 2023-08-24

**Authors:** Lars Masanneck, Jan Voth, Niklas Huntemann, Menekse Öztürk, Christina B. Schroeter, Tobias Ruck, Sven G. Meuth, Marc Pawlitzki

**Affiliations:** 1 Department of Neurology, Medical Faculty University Hospital Düsseldorf, Heinrich-Heine University Duesseldorf, Moorenstraße 5, 40225 Düsseldorf, Germany; 2 Hasso Plattner Institute, University of Potsdam, 14482 Potsdam, Germany

**Keywords:** Clinical trial, Digital health technology, CIDP, End-of-dose, Smartwatch

## Abstract

**Introduction:**

Chronic inflammatory demyelinating polyneuropathy (CIDP) is one of the most common immune neuropathies leading to severe impairments in daily life. Current treatment options include intravenous immunoglobulins (IVIG), which are administered at intervals of 4–12 weeks. Determination of individual treatment intervals is challenging since existing clinical scores lack sensitivity to objectify small, partially fluctuating deficits in patients. End-of-dose phenomena described by patients, manifested by increased fatigue and worsening of (motor) symptoms, are currently difficult to detect. From a medical and socio-economic point of view, it is necessary to identify and validate new, more sensitive outcome measures for accurate mapping of disease progression and, thus, for interval finding. Digital health technologies such as wearables may be particularly useful for this purpose, as they record real-life data and consequently, in contrast to classic clinical 'snapshots’, can continuously depict the disease course.

**Methods:**

In this prospective, observational, non-interventional, single-center, investigator-initiated study, CIDP patients treated with IVIG will be continuously monitored over a period of 6 months. Clinical scores and blood analyses will be assessed and collected during three visits (V1, V2, V3). Additionally, activity, sleep, and cardiac parameters will be recorded over the entire period using a wearable device. Further, patients' subjective disease development and quality of life will be recorded at various visits (read-outs). The usability of the smartwatch will be assessed at the end of the study.

**Perspective:**

The study aims to evaluate different digital measurements obtained with the smartwatch and blood-based analyses for monitoring disease activity and progress in CIDP patients. In conjunction, both means of monitoring might offer detailed insights into behavioral and biological patterns associated with treatment-related fluctuations such as end-of-dose phenomena.

***Trial registration*:**

The study protocol was registered at ClinicalTrials.gov. Identifier: NCT05723848. Initially, the protocol was submitted prospectively on January 10, 2023. The trial was publicly released after formal improvements on February 13, 2023, after first patients were included according to the original protocol.

## Introduction

Chronic inflammatory demyelinating polyneuropathy (CIDP) is one of the most common immune neuropathies [11]. It is characterized by a progressive or relapsing disease course developing for more than 8 weeks, often leading to severe restriction of the patient's everyday life [5, 14]. Typical symptoms are symmetrical distal and proximal paresis, sensory disturbances, and reduced to absent reflexes. Therapeutic long-term options, in addition to glucocorticoids and plasma exchange, are mainly intravenous immunoglobulins (IVIG) (Van den [16], which must be administered repeatedly to ensure disease stability [9]. As typical intervals between IVIG applications range from 4 to 12 weeks, individual interval finding is sometimes difficult in the context of CIDP. In daily routine, treatment intervals primarily rely on clinical examinations, which frequently struggle to quantify patients' fluctuating deficits effectively. Alternatively, slight changes relevant to patients’ everyday lives are sometimes taken as indicators and lead to alterations of the individual dosing interval. Therefore, the subjective perspective of patients is even more relevant for the adjustment of the therapy intervals.

Moreover, some CIDP patients undergoing IVIG treatment often describe treatment-related fluctuations or an "end-of-dose" phenomenon at the end of their respective treatment cycle, which is characterized by increased fatigue and, eventually, deterioration of pre-existing neurological deficits [2, 3]. In addition, patients frequently report significant improvement shortly after the infusion, a phenomenon not yet explained from a pathophysiological standpoint. These gradual changes and fluctuations cannot be adequately reflected by current measurements, which only offer a ‘snapshot’ of the patient’s status at the time and place of examination [7]. Moreover, current outcome measures from prospective trials in IVIG treated patients do not allow meaningful conclusions for daily routine [1]. Consequently, dosing intervals today are usually based on insufficient information, as objectifiable parameters such as nerve conduction studies are neither dynamic nor sensitive enough to document the changing patient’s everyday limitations [2]. Since IVIG treatment must be critically reviewed from a medical and socio-economic point of view (availability, potential side effects, high costs) [4], new markers for disease activity are needed to monitor the patient’s status and optimize treatment continuously. Although there are initial attempts to more closely monitor the clinical development of patients [2], these require daily clinical measurements and are currently not routinely used.

Digital Health Technologies (DHTs), like connected wearables [6], provide nearly continuous real-life data collection with unparalleled frequency, allowing researchers and clinicians to transition from 'snapshot' assessments to more dynamic evaluations of patients' status [15]. As DHTs can increasingly document movement patterns, sleep behavior, and cognition, their use in neurology trials continues to grow [12]. Together with conventional measurements, this ‘digital phenotyping’ might lead to a more complete and continuous recording of deficits relevant to everyday life. Given the high need to better document fluctuating disability in CIDP patients, DHTs might both enable a better understanding of disease activity for individual patients and assist clinicians in drawing relevant therapeutic decisions for the individual CIDP patient in a precision-medicine-guided manner.

## Methods

### Aim of the trial

The goal of the EMDA CIDP study is to evaluate various widely available smartwatch-derived digital metrics and blood-based analyses for monitoring disease activity in CIDP patients treated with IVIG.

First, digital and blood-based measurements will be compared with subjective patient reports and established clinical scores. Second, exploratory analyses aim to understand longitudinal disease progression and its variability in individual patients. Thus, by monitoring a variety of digital and biological phenotypes of CIDP patients, individual determinants and measurements of disease activity patterns will be explored and characterized. Novel auspicious combinations of disease activity measurements may improve dose and interval optimization in the future. Considering the influence of behavioral traits such as physical activity and sleep on patients' metabolic state and various bodily functions [8, 10], we aim to explore the interplay between behavioral and biomedical characteristics in our patients. By monitoring both behavioral and biomedical parameters over time, we further aim to investigate the potential response of biomedical features to changes in activity or sleep patterns.

### Study description and study design

This prospective, observational, non-interventional, single-center, investigator-initiated study is being conducted at the University Hospital of Düsseldorf, Germany.

**Fig. 1:**
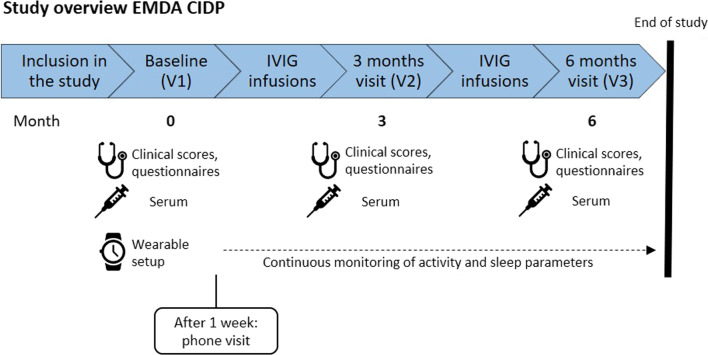
Graphical outline of the study protocol. *IVIG* intravenous immunoglobulins

Upon introduction to the trial and written consent, CIDP patients treated with IVIG are enrolled in the study during the screening visit in conjunction with the baseline visit (V1). During this onsite visit participants are handed out a wearable (ScanWatch, Withings, Paris, France) and instructed in the use of the technology. After a 1-week period, a phone visit will be conducted to clarify any technical issues. Wearables are used to continuously record patients' activity, cardiac, and sleep parameters for a total of 6 months. In addition, a second onsite visit (V2) is scheduled after 3 months, and a final visit (V3) after 6 months. At all three onsite visits, clinical examinations, scores to classify CIDP-related symptom severity, and questionnaires regarding quality of life, quality of sleep, and the subjective occurrence of end-of-dose phenomena will be collected. In addition, a serum sample will be collected to analyze markers of neuroaxonal damage such as serum neurofilament light chain (sNfL). Furthermore, we aim to employ non-targeted proteomics to systematically analyze protein expression patterns in CIDP patient samples, aiming to identify potential biomarkers or therapeutic targets. By combining this comprehensive proteomic data with digitally captured behavioral information, we seek to uncover novel insights into proteomic-behavioral interactions in CIDP patients, ultimately improving disease understanding and facilitating personalized treatment strategies (see Fig. 1).

Throughout the period, patients will receive their regularly scheduled IVIG infusions.

Sample size calculation: Given the exploratory and observational nature of our study, we do not have a specific hypothesis to test, making power analysis less suitable in this context where basic information on distributions is currently missing. The primary objective is to generate first-hand data to enable hypothesis formation and identify potential relationships for further examination in subsequent hypothesis-driven studies.

### Arms and interventions

As this is an observational cohort study, there are neither interventions nor arms. Patients will be stratified by their biological and digital behavioral phenotypes retrospectively to assess differences in disease activity patterns.

It is planned to enroll approximately 60 patients in the study.

### Outcome measures

The following clinical scores and questionnaires, as well as blood analyses, will be collected at all three visits (V1, V2, V3) and compared over time:

Clinical scores:

- Inflammatory Rasch-built Overall Disability Scale (I-RODS)
- Inflammatory Neuropathy Cause and Treatment (INCAT) disability score
- Grip strength
- Medical Research Council (MRC)-Sumscore

Questionnaires:

- Subjective occurrence of end-of-dose phenomena/wearing off
- Abbreviated World Health Organization Quality of Life (WHOQOL-BREF)
- Pittsburgh Sleep Quality Index (PSQI)

Blood analyses:

- Levels of sNfL
- Serum proteomics and metabolomics

A questionnaire on smartwatch use containing the System Usability Scale will additionally be asked at the final visit (V3).

Over the entire period of 6 months, the following outcome measurements will be recorded with the Withings ScanWatch:

- Wearing time of smartwatch (daily)
- Longitudinal development of activity parameter: step count, approximate distance traveled (meter), duration of soft, moderate, and intense activity (seconds) defined by Withings, the sum of all active time (seconds), approximate calories burned
- Longitudinal development of sleep parameter: time awake (seconds), number of times user woke up, time to sleep (seconds), total time in bed (seconds), total time asleep (seconds), ratio of sleep/ time in bed, time spent in bed before falling asleep (seconds), time awake after first falling asleep (seconds), Withings Sleep score defined by Withings
- Longitudinal development of cardiovascular parameters: average heart rate, maximal heart rate, minimum heart rate, time in light, moderate, intense, and maximal heart rate zone (seconds) defined by Withings
- Light heart rate zone: from 0% inclusive to 50% exclusive of maximum heart rate. Moderate heart rate zone: from 50% included to 70% excluded of maximal heart rate. Intense heart rate zone: from 70% included to 90% excluded of maximal heart rate. Maximal heart rate zone: from 90% included to 100% included of maximal heart rate.
- Number and time of irregular 1-channel ECGs (according to Withings algorithm)

### Eligibility criteria

CIDP patients treated with IVIG in constant intervals are included.

Further inclusion criteria are:

- Age ≥ 18 years
- Probable or definite CIDP patients according to the European Federation of Neurological Societies/Peripheral Nerve Society 2010 criteria [16].
- IVIG treated as follows:
  1. Documented evidence of objective response to IVIG, with clinically meaningful improvement. Clinically meaningful improvement is defined as one of the following: ≥ 1 point decrease in adjusted INCAT score, ≥ 4 points increase in I-RODS total score, ≥ 3 points increase in MRC Sum score, ≥ 8 kilopascal improvement in mean grip strength (one hand), or an equivalent improvement based on information documented in medical records and per the principal investigator's (PI) judgement.
  2. Must be on stable IVIG therapy, defined as no change greater than 10% in frequency or dose of immunoglobulin therapy or corticosteroids within 8 weeks prior to screening.
  3. Evidence of clinically meaningful deterioration on interruption or dose reduction of IVIG therapy within 24 months prior to screening, determined by clinical examination or medical records. Clinically meaningful deterioration is defined as one of the following: ≥ 1 point increase in adjusted INCAT score, decrease in I-RODS total score ≥ 4 points, decrease in MRC Sum score ≥ 3, mean grip strength worsening of ≥ 8 kilopascals (one hand), or an equivalent deterioration based on information from medical records and at the PI's judgement.

The exclusion criteria are:

- unable to use smartwatch or/ and smartphone device

To maximize recruitment potential in a rare disease and minimize bias in this real-world measurement cohort, other comorbidities are not considered causes for exclusions. While this might be a source of potential error, we assess patients at every visit for comorbidities or special life events to best correct for effects on intra-patient measurements.

### Laboratory methods

- sNfL: we plan to use a single molecule array with a SiMoA HD-1 (Quanterix, USA) using the Nf-Light Advantage Kits (Quanterix) according to manufacturer’s instructions for NfL measurements to measure sNfL in sera obtained at each visit of patients.
- Proteomics: Serum samples are planned to be depleted using the ProteoMiner kit (Bio-Rad Laboratories Inc., Hercules, CA, USA) according to the manufacturer’s instructions. After further standard processing steps such as reduction and alkylation, peptides will be collected, dried and resolved. Peptide solutions are set to be analyzed by reversed-phase chromatography coupled to ion mobility mass spectrometry with Synapt G2 Si/ M-Class nano-ultra performance liquid chromatography (UPLC) (Waters Corporation, Milford, MA, US). Analysis is planned to be carried out using Progenesis for Proteomics (Walters) and Perseus.
- Metabolomics: The exact protocol is currently under development, we aim to integrate serum sample preprocessing as an alternative to above-mentioned processing. The current goal is to base the metabolomics analysis on mass spectrometry as well.

### Analysis strategy

The planned study is intended as an exploratory study, i.e. all results will be interpreted as hypotheses generating. We will correlate clinical, digital and proteomic parameters as well as markers of neuroaxonal damage at baseline and with regard to longitudinal changes. Exemplary analyses will include:

- Advanced machine learning algorithms, employing both linear and non-linear boosted regression, along with gradient boosted random forest trees and potentially other machine learning algorithms, will be implemented for integrative prediction and categorization of clinical parameters, using biomedical (inflammation markers, proteome, metabolome) data as the base.
- Data of the different digitally tracked features will be analyzed using time series analysis methods and will be screened for trends, autocorrelation and seasonality components as well as feature analysis and time series decomposition. Features and traits of this time series data will be correlated with clinical developments as well as with biological features such as promisingmarkers like sNfL or proteomic marker candidates.
- Aggregated digital measures of different timepoints in the treatment cycle will be compared repetitively with each other (e.g. first week after treatment vs. week before treatment) and screened for differences on an individual and a group level, which might point to end-of-dose phenomena. Further stratification might include subgroups based on clinical or biomedical features.
- Repetitive trends in digitally-acquired time series data will be analyzed and potentially modeled using time-split data, deploying autoregressive moving average models as well as other algorithms.

### Contacts

The EMDA CIDP was initiated at the University Hospital Düsseldorf by the Department of Neurology under the direction of Prof. Dr. med. Dr. rer. nat. Dr. h.c. Sven Meuth. The PI is PD Dr. med. Marc Pawlitzki, senior physician.

## Perspective

**Fig. 2:**
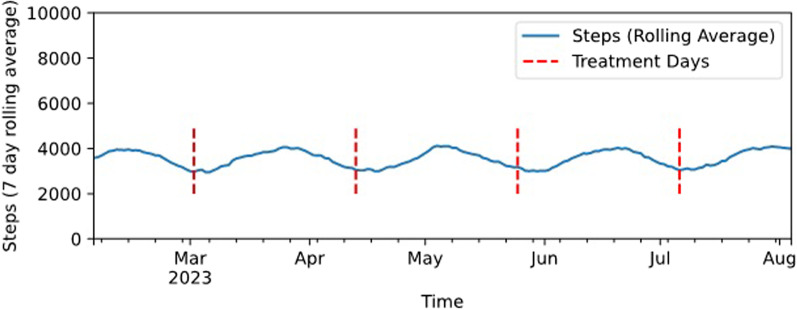
Hypothetical simulated activity course of a study patient. The simulated graph shows a hypothetical example of the 7 day rolling average of step counts for a study patient over the course of 6 months with an intravenous immunoglobulins (IVIG) treatment interval of 6 weeks. One hypothesis this study aims to clarify is whether smartwatch-obtained high-frequency measurements might be able to reflect patient-reported end-of-dose phenomena or treatment-related fluctuations. Shortly after the administration of IVIG, the patient's activity might initially increase until it drops again later in the treatment cycle

The EMDA CIDP trial aims to capture both digital behavioral data and biological markers to assess disease activity and fluctuations. Comprehensive biological assessments of neurological damage and serum analytics provide the basis for patient cohort stratification and biomarker exploration. Capturing a detailed digital phenotype of patient activity and other data might enable observations of fluctuations in behavior in accord with the subjective end-of-dose phenomenon (see Fig. 2). Additionally, such longitudinal high-frequency data might help to better understand the individual disease activity and reflect disease-related deficits in the daily life of CIDP patients. In combination, digital and biological means of monitoring might offer detailed insights into behavioral and biological patterns that correlate with disease activity or progress.

Potential markers for disease fluctuations may be used to personalize treatment and dosing intervals in future studies. Digital disease monitoring may also be extended outside sparse specialized centers [13], potentially improving monitoring and healthcare access in rural regions alike. Additionally, a better understanding of contributing factors to disease activity may lead to a better understanding of the pathophysiological processes underlying CIDP.

## Data Availability

Not applicable.

## Abbreviations

EMDA: Electronic Monitoring of Disease Activity
CIDP: Chronic inflammatory demyelinating polyneuropathy
DHTs: Digital health technologies
INCAT: Inflammatory Neuropathy Cause and Treatment
I-RODS: Inflammatory Rasch-built Overall Disability
IVIG: Intravenous immunoglobulins
MRC: Medical Research Council
PI: Principal investigator
PSQI: Pittsburgh Sleep Quality Index
sNfL: Serum neurofilament light chain
WHOQOL-BREF: Abbreviated World Health Organization Quality of Life
V1: Baseline visit
V2: 3 Months visit
V3: Final visit
ECG: Electrocardiography
